# Deep Cleaning Device as a Treatment Is Effective for Blepharitis and Significantly Reduces Bacterial Load

**DOI:** 10.1155/joph/1518139

**Published:** 2026-06-02

**Authors:** Ya Wen, Jiayu Bao, Ao Li, Yubo Wu, Yang Zhang, Lei Tian, Ying Jie

**Affiliations:** ^1^ Beijing Tongren Hospital, Capital Medical University, Beijing, 100730, China, ccmu.edu.cn; ^2^ Beijing Tongren Eye Center, Beijing, 100730, China; ^3^ Beijing Institute of Ophthalmology, Beijing, 100730, China; ^4^ Beijing Ophthalmology & Visual Sciences Key Laboratory, Beijing, 100730, China, ccmu.edu.cn; ^5^ School of Basic Medical Sciences, Capital Medical University, Beijing, 100069, China, ccmu.edu.cn

**Keywords:** bacterial load, blepharitis, deep cleaning device, ocular surface health, treatment

## Abstract

**Objective:**

To evaluate the efficacy and safety of the Deep Cleaning Device for the treatment of blepharitis.

**Methods:**

This prospective, randomized controlled trial enrolled 51 adult patients with blepharitis, who were randomly allocated to two treatment groups. Group A (*n* = 26) received a combined therapy of sodium hyaluronate eye drops plus weekly device‐assisted eyelid margin cleaning, while Group B (*n* = 25) received sodium hyaluronate eye drops as monotherapy. The primary efficacy indices included subjective symptoms, bacterial load of eyelid margin, and eyelid margin scale and hyperemia grading. Secondary efficacy indices covered meibomian gland expressibility, meibum quality, tear film stability, corneal fluorescein staining score, tear meniscus height, and lipid layer thickness. Safety indicators include best‐corrected visual acuity, noninvasive intraocular pressure, and adverse events.

**Results:**

Group A exhibited significant improvements in reducing bacterial load and alleviating subjective symptoms compared to Group B. Objective signs, including eyelid margin scale grading and meibum quality score, also showed marked improvement. Safety assessments confirmed no significant changes in best‐corrected visual acuity or intraocular pressure. No patient reported serious adverse reactions.

**Conclusions:**

The Deep Cleaning Device is a safe and effective treatment for blepharitis, significantly reducing bacterial load and alleviating clinical symptoms. It represents a valuable addition to conventional therapies.

**Trial Registration:** Chinese Clinical Trial Registry: ChiCTR2200057855.

## 1. Introduction

Blepharitis is a highly prevalent and etiologically complex chronic ophthalmic disease that affects individuals across all age groups [1]. In ophthalmic practice, it is a common presentation, often presenting with pronounced ocular symptoms, such as eyelid margin erythema, scaling, itching, and dry eye, all of which can significantly impair the quality of life [2]. Clinical examination may reveal the presence of scales on the eyelid margins, telangiectasia, conjunctival hyperemia, punctuate keratopathy, cornea vascularization, and ulceration. Individuals suffering from long‐standing chronic blepharitis may exhibit a range of complications, including eyelid margin hypertrophy, scarring, eyelash loss, and trichiasis [3]. Among patients experiencing recurrent episodes, particularly children and adolescents, blepharokeratoconjunctivitis is a frequent occurrence. This condition can result in chronic eyelid inflammation and may trigger a range of complications, including corneal astigmatism, corneal pathology, amblyopia, vision loss, and in severe cases, corneal perforation [4].

According to the 2024 Blepharitis Preferred Practice Pattern [1], blepharitis can be divided into anterior (affecting eyelid skin, lash base, and follicles) and posterior (involving meibomian glands) types. Traditionally, blepharitis has been clinically classified as staphylococcal, seborrheic, meibomian gland dysfunction (MGD), or a combination of both. Staphylococcal blepharitis is associated with an increased local load of *Staphylococcus aureus* [5]. Seborrheic blepharitis is often associated with seborrheic dermatitis and may also be accompanied by an increase in local bacterial load [6, 7]. With the continuous advancement of technology, research on the ocular surface microbiota and its impact on ocular surface inflammation has deepened significantly [8, 9]. Multiple studies have shown that an increase in local bacterial load and alterations in the composition of the microbiota may be important factors for the recurrence of blepharitis and its long‐term impact on patients’ lives [10–13]. Compared with healthy controls, patients with blepharitis had significantly higher bacterial positive culture percentage and colony number [10, 14]. Studies examining the composition and diversity of the ocular surface microbiota in blepharitis patients demonstrated distinct differences in the relative abundance of bacterial species within the ocular surface flora of blepharitis patients compared to healthy individuals [11, 13]. This implies a close link between the balance or symbiosis of the ocular microbiome and blepharitis severity. Therefore, for blepharitis patients with ocular surface microbiome imbalance, reduction of the bacterial load may be crucial for disease improvement.

Blepharitis is characterized by recurrent attacks, thus requiring persistent and comprehensive management. Currently, in addition to conventional prescription ophthalmic medications such as artificial tears, nonsteroidal anti‐inflammatory drugs (NSAIDs), corticosteroids, and antibiotics [15], ongoing daily practices, such as eyelid hygiene, warm compresses, and meibomian gland massage, are equally important and have been incorporated into the treatment protocols for blepharitis to reduce the risk of recurrence [16]. Proper cleansing of the eyelids and eyelid margins is crucial for reducing the load of harmful bacteria on the eyelid margin, which can effectively relieve the symptoms and signs of blepharitis. Consequently, there has been a surge in the development and clinical application of eyelid cleansing products in recent years [17, 18]. Beyond the earliest used baby shampoo, specialized ocular cleansers or wipes containing antibacterial or anti‐mite ingredients (e.g., tea tree oil and hypochlorous acid) are available [19, 20]. These ingredients are nonirritating and safe. Their ease of operation allows patients to conveniently apply them at home [17]. However, if patients lack the manual dexterity or necessary skills to perform the task safely, it can be problematic. Furthermore, daily eyelid hygiene demands high patient compliance; failure to adhere to a regular regimen can lead to disease recurrence. Currently, several in‐office procedural treatments have been developed to standardize eyelid cleaning. BlephEx™ Microblepharo Exfoliation can effectively reduce the number of *Demodex* mites in patients with *Demodex* blepharitis, improve subjective symptoms, and be helpful for treatment [21, 22]. In addition, it can reduce the number, separation frequency, and ratio of Gram‐positive rod cells and cocci at the eyelid margin and improve the eyelid signs and tear film characteristics of symptomatic contact lens wearers [23, 24]. BlephEx™ Microblepharo Exfoliation was also used in the treatment of chalazia and has been found to effectively promote the regression of chalazia in patients [25]. Another Deep Cleaning Device can improve the subjective symptoms of patients with MGD‐associated dry eye and prolong the tear film breakup time [26]. A novel eye brush was also used for eyelid margin cleaning and is more effective than using cleaning liquid alone [27]. Similarly, in‐office procedural treatments also have inherent limitations, including the need for regular clinic visits, associated costs, and potential accessibility issues for patients in remote areas or with limited mobility. Although the effectiveness of the in‐office procedural treatments for eyelid margin diseases has been confirmed, it remains unclear whether they can effectively reduce the bacterial load of patients with blepharitis and improve symptoms and signs. This study aimed to evaluate the efficacy of the newly developed Deep Cleaning Device (Ocuface Medical Co., Ltd., Guangzhou, China) as a treatment for blepharitis, focusing on its impact on bacterial load and clinical symptoms and signs.

## 2. Methods

### 2.1. Study Design

This single‐center, prospective, randomized controlled study enrolled adult patients with blepharitis in the Ophthalmology Department of Beijing Tongren Hospital, Capital Medical University. Randomization was performed via computer‐generated sequence with allocation concealment achieved using opaque envelopes. To maintain blinding integrity, outcome assessors were masked to treatment allocation throughout the study. An independent clinician team was implemented, with one group exclusively administering interventions and another distinct group conducting all clinical evaluations, thereby ensuring objective assessment. This study complied with the principles of the Declaration of Helsinki and was approved by the Ethics Review Committee of Beijing Tongren Hospital (Ethics Approval Number: TRECKY2021‐065). All enrolled patients provided written informed consent using informed consent approved by an institutional review board. This study was enlisted in the Chinese Clinical Trial Registry.

### 2.2. Participants

A total of 51 patients with blepharitis (15 males and 36 females) were enrolled in this study between June 2022 and October 2023. All participants met the following inclusion and exclusion criteria. One eye of each patient was chosen as the study eye. If both eyes met the inclusion criteria, the eye with the higher score of signs at the screening visit was considered the analysis eye; if both eyes had equal score, the right eye was the analysis eye.

#### 2.2.1. Inclusion Criteria

Inclusion criteria were as follows: aged ≥ 18 years; presence of ocular discomfort symptoms (itching, foreign body sensation, and burning); and clinical signs of blepharitis (scales and meibomian gland capping or collarettes).

#### 2.2.2. Exclusion Criteria

Exclusion criteria included the following: systemic or topical antibiotic use within 4 weeks prior to enrollment; current use of dry eye therapies or lid hygiene regimens (including warm compresses, ocular cleansers, or wipes); regular contact lens wear (daily use); presence of other eyelid or ocular surface diseases; recent (≤ 6 months) ocular surgery or trauma affecting corneal sensitivity/tear film; pregnancy or lactation; autoimmune diseases (e.g., rheumatologic conjunctivitis, Sjögren’s syndrome, and endocrine orbitopathy); presence of trachoma; and severe systemic diseases.

### 2.3. Interventions

#### 2.3.1. Treatment Protocol

**FIGURE 1:**
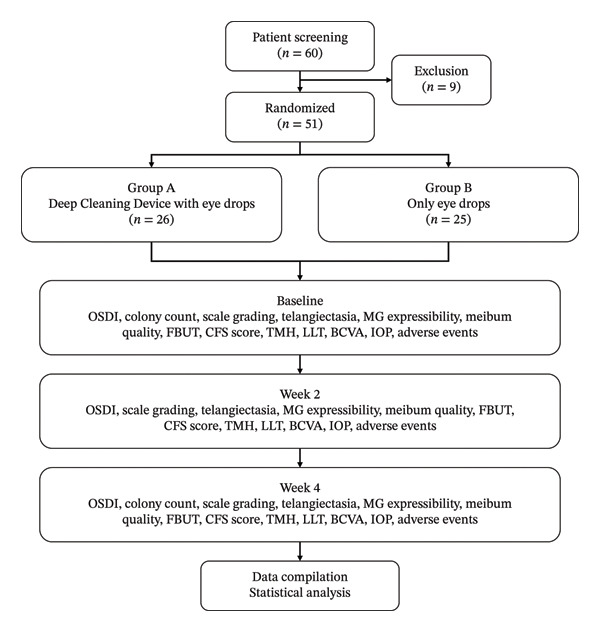
Study flowchart. This study was structured into four distinct phases to ensure comprehensive data collection and analysis: the prescreening phase, treatment initiation on baseline, and follow‐up assessments at Weeks 2 and 4. Throughout each phase, patient data were systematically gathered in accordance with the predefined study protocol.

The screening process is shown in Figure 1. At the initial clinic visit, patients with blepharitis were screened by the attending physician according to the inclusion and exclusion criteria. If patients met all study criteria, they were informed about the trial and provided with a consent form. The patients were then randomly assigned to one of two groups based on the randomization numbers generated using Microsoft® Office Excel 2020. Group A: the participants were treated with 0.3% sodium hyaluronate eye drops four times a day (Santen Pharmaceutical Co., Ltd.) in combination with deep cleaning of eyelid margin once per week. Group B: the participants were treated with only sodium hyaluronate eye drops. Tobramycin dexamethasone eye ointment was administered during the first week as a standardized initial anti‐inflammatory and antibacterial treatment to control acute inflammation and provide symptomatic relief, following common clinical practice for blepharitis management [1]. This 1‐week course was applied uniformly to both groups to establish a comparable baseline before evaluating the specific effects of the deep cleaning intervention. Follow‐ups were performed at Weeks 2 and 4 after random assignment. All cleaning treatments were carried out by the same doctor, and subsequent observations and data collection were carried out by another trained masked doctor.

#### 2.3.2. Deep Cleaning Treatment

**FIGURE 2:**
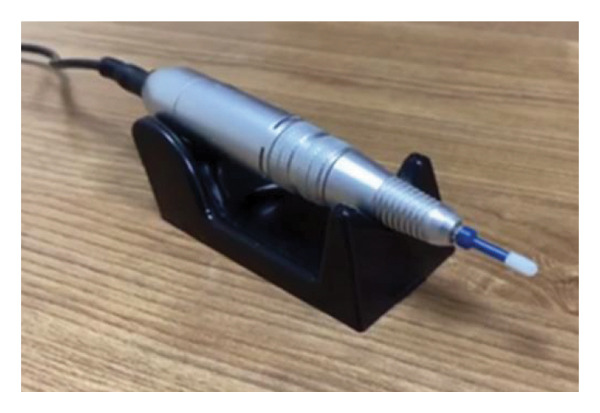
The Deep Cleaning Device. The Deep Cleaning Device comprises an electric cleaning handle and a brush head. During operation, grasp the handle as would a pen and utilize the high‐speed rotating brush head to meticulously clean the eyelid margin.

The Deep Cleaning Device consisted of an electric cleaning handle and a brush head (Figure 2). The brush head was disposable and made of sterile sponge. The system dynamically regulated brush head rotation speed (range: 0–4500 rpm) according to real‐time assessments of eyelid debris removal efficacy and subjective patient tolerance.

Before the procedure, the periocular skin was prepared by aseptic cleansing with an electrolyzed water–sodium chloride solution. The installed brush head was thoroughly soaked with the same solution. The patient was placed on the examination bed, and after local anesthesia with proparacaine hydrochloride eye drops, the eyelid skin was gently pushed with a cotton swab to expose the eyelid margin. The instrument was activated, and the moistened brush head was applied perpendicularly to the eyelid margin. A constant‐speed motion was maintained from the nasal to temporal aspect for 15–20 s per pass, with this procedure repeated 2–4 times per treatment session. The lash base was subsequently cleansed using the same standardized protocol. Patients were instructed to direct their gaze accordingly: downward for upper lid treatment and upward for lower lid treatment. Finally, the treated areas were gently wiped with 0.9% sodium chloride–soaked gauze pads.

### 2.4. Outcome Measures

#### 2.4.1. Primary Efficacy Index

##### 2.4.1.1. Subjective Symptoms

During each follow‐up, all participants were required to complete the Ocular Surface Disease Index (OSDI) questionnaire [28]. The OSDI questionnaire consisted of 12 questions, with a total score of 0–100, and assessed the severity of subjective symptoms from three aspects: ocular symptoms, visual function, and environmental triggers. The higher the score, the more severe the symptoms.

##### 2.4.1.2. Bacterial Culture and Colony Count

A sterile cotton swab was used to wipe the eyelid margin of the patient and dissolved in 1 mL phosphate‐buffered saline (PBS) to make a suspension solution. The bacterial suspension was transferred to the blood agar plate culture medium for 24 h, and the colonies formed on the plate were counted (CFU/mL). The colony count quantitative experiment was conducted before treatment and 1 month after treatment.

##### 2.4.1.3. Eyelid Margin Scaling Grade

Under slit lamp, the scale distribution of eyelid margins was measured, respectively, in upper and lower eyelids. Based on the extent of the involved area, eyelid margin scaling was classified into four grades: Grade 0 = no eyelid margin scale; Grade 1 = eyelid margin scales spread over less than 1/3 of the margin; Grade 2 = scales spread over 1/3 to 2/3 of the eyelid margin; and Grade 3 = eyelid margin scales spread over more than 2/3 of the margin.

##### 2.4.1.4. Eyelid Margin Telangiectasia

The distribution of eyelid margin vessels was observed under slit lamp. The grading scale reported by Arita et al. was used to assess eyelid margin involvement in a blinded manner based on the eyelid margin photos before and after treatment [29]: Grade 0 = no findings; Grade 1 = mild telangiectasia; Grade 2 = moderate telangiectasia or redness; and Grade 3 = severe telangiectasia or redness.

#### 2.4.2. Secondary Efficacy Index

##### 2.4.2.1. Meibomian Gland Expressibility

The central five glands of the lower eyelids were pressed with Meibomian Gland Evaluator (MGE; Tear Science, Inc., Milpitas, USA), and the amount of meibum extruded was observed to judge its expressibility [30]. Meibomian gland expressibility was thought to reflect meibomian gland function. The scoring criteria were as follows: Grade 0 = secretion is extruded from all glands; Grade 1 = 3 ∼ 4‐gland secretion extrusion; Grade 2 = 1 ∼ 2‐gland secretion extrusion; and Grade 3 = no secretion extruded from all glands.

##### 2.4.2.2. Meibum Quality (MQ) Score

Meibum was extracted from the central five meibomian glands by gently pressing 1‐2 mm below the eyelid margin. The properties of meibum were observed and scored according to a previously proposed method [30]. Five central glands of the upper and lower eyelids were evaluated. Grade 0 = clear fluid; Grade 1 = cloudy fluid; Grade 2 = cloudy particulate fluid; and Grade 3 = inspissated, toothpaste‐like discharge.

##### 2.4.2.3. Fluorescein Tear Film Breakup Time (FBUT)

After applying a wet sterile fluorescein strip to the lower fornix, the time interval between the last blink and the appearance of the first random dry spot on the corneal surface was recorded using a cobalt blue filter slit lamp, which is known as the FBUT.

##### 2.4.2.4. Corneal Fluorescein Staining (CFS) Score

The CFS score was assessed under cobalt blue light using the American National Eye Institute/Industry (NEI) scale (total score of 0–15) to evaluate the severity of ocular surface damage after staining.

##### 2.4.2.5. Tear Meniscus Height (TMH)

The white light tool of German OCULUS 5M corneal topographer (K5M) was used to measure the TMH image of the patient. TMH was formed by the tear gathered at the upper and lower eyelid margin, representing the volume of tear secretion and reflecting lacrimal gland function. The central TMH data of the lower eyelid were acquired by the measuring tool in the system and recorded in millimeters (mm).

##### 2.4.2.6. Lipid Layer Thickness (LLT)

The LipiView interferometer (TearScience, Morrisville, USA) analyzed the LLT based on a 20‐second video. LLT reflected the ability of meibomian gland to secrete lipids.

#### 2.4.3. Safety Assessment

Comprehensive safety monitoring was conducted at all follow‐up visits, including evaluation of best‐corrected visual acuity (BCVA), noninvasive intraocular pressure (IOP) measurement, and detailed anterior segment examination using slit lamp. Meanwhile, the feelings and adverse events of the patients during and after the treatment were recorded. These evaluations were performed by two experienced ophthalmologists from Beijing Tongren Hospital.

### 2.5. Sample Size

As this was an exploratory study without prior knowledge of effect sizes or variances, a priori sample size calculation was not feasible.

### 2.6. Statistical Analysis

All statistical analysis was conducted using SPSS 27.0 (SPSS Inc., USA). Chi‐square test was used to analyze the difference of sex ratio between the two groups. Data with a normal distribution were presented as mean ± standard deviation. Independent sample *t* test was used to analyze the baseline difference between the two groups. Univariate repeated measure analysis of variance (ANOVA) was used for the overall comparison before and after treatment, and LSD‐t test was used for comparison between pair and pair time points. Nonnormally distributed data were presented as median (first quartile, third quartile); Mann–Whitney U rank‐sum test was used for baseline difference analysis between the two groups, linear mixed‐effects model was used for overall comparison before and after treatment, and Wilcoxon rank‐sum test was used for comparison at two time points. A two‐sided test with *p* < 0.05 was considered to indicate statistical significance.

## 3. Results

### 3.1. Baseline Data

A total of 51 participants with blepharitis were enrolled and completed the study. The detailed description of the baseline data of the participants is portrayed in Table 1. There were no significant differences between the two groups in the baseline test results including gender, age, and primary and secondary efficacy index.

**TABLE 1:** Baseline demographic and clinical characteristics of each group.

Characteristics	Group A	Group B	*p* value
Participant (*n*)	26	25	
Gender (male/female, *n*)	8/18	7/18	0.828
Age (years)	35.27 ± 14.12	38.56 ± 13.53	0.400
OSDI score	46.90 ± 17.80	51.06 ± 22.91	0.472
Colony count (CFU/mL)	114.81 ± 60.65	103.84 ± 41.50	0.456
Scale grading (upper eyelid)	1.5 (1, 2)	2 (1, 2)	0.893
Scale grading (lower eyelid)	1.5 (1, 2)	1 (1, 2)	0.728
Telangiectasia (upper eyelid)	2 (1, 2)	1 (1, 2)	0.586
Telangiectasia (lower eyelid)	2 (1, 2)	1 (1.2)	0.281
Meibomian gland expressibility (upper eyelid)	1 (1, 2)	1 (1, 2)	0.493
Meibomian gland expressibility (lower eyelid)	1 (1, 2)	1 (1, 2)	0.556
MQ score (upper eyelid)	2 (1, 2)	2 (2, 2)	0.080
MQ score (lower eyelid)	1 (1, 1)	2 (1, 2)	0.203
FBUT (s)	3.53 ± 1.74	3.70 ± 1.63	0.731
CFS score	2.92 ± 1.26	2.80 ± 1.08	0.710
TMH (mm)	0.19 ± 0.06	0.23 ± 0.12	0.074
LLT (nm)	75.15 ± 22.32	80.20 ± 21.19	0.412
BCVA	0.66 ± 0.36	0.63 ± 0.33	0.777
IOP (mmHg)	12.62 ± 2.54	12.94 ± 2.27	0.633

*Note:* FBUT = fluorescein tear film breakup time; IOP = noninvasive intraocular pressure.

Abbreviations: BCVA = best‐corrected visual acuity; CFS = corneal fluorescein staining; LLT = lipid layer thickness; MQ = meibum quality; OSDI = Ocular Surface Disease Index; TMH = tear meniscus height.

### 3.2. Primary Efficacy Index

Overall, there were statistically significant improvements in OSDI score, colony count, and scale grading after treatment in both groups. Both groups exhibited improvements over time. As the duration of treatment increased, the differences between the treatment group and the control group became more pronounced. However, no statistical significance was detected in the telangiectasia (Table 2 and Figure S1).

**TABLE 2:** The primary efficacy index before and after treatment.

Index	Group	Baseline	Week 2	Week 4	*p* ^∗^
OSDI score	A	46.90 ± 17.80	32.03 ± 17.49^a^	20.75 ± 15.62^ab^	< 0.001
	B	51.06 ± 22.91	40.19 ± 16.89	31.23 ± 18.49^a^	< 0.001
	*p* ^#^	0.472	0.097	0.033	

Colony count (CFU/mL)	A	114.81 ± 60.65	—	4.58 ± 3.57^a^	< 0.001
	B	103.84 ± 41.50	—	9.12 ± 8.33^a^	< 0.001
	*p* ^#^	0.456	—	0.017	

Scale grading (upper eyelid)	A	1.5 (1, 2)	1 (0, 1)^a^	0 (0, 0)^ab^	< 0.001
	B	2 (1, 2)	1 (1, 2)^a^	0 (0, 1)^ab^	< 0.001
	*p* ^#^	0.893	0.046	0.043	

Scale grading (lower eyelid)	A	1.5 (1, 2)	1 (1, 1)^a^	0 (0, 0)^ab^	< 0.001
	B	1 (1, 2)	1 (1, 1)^a^	1 (0, 1)^ab^	< 0.001
	*p* ^#^	0.728	0.024	< 0.001	

Telangiectasia (upper eyelid)	A	2 (1, 2)	1 (1, 1)	1 (1, 1)	0.080
	B	1 (1, 2)	1 (1, 2)	1 (1, 2)	0.251
	*p* ^#^	0.586	0.184	0.107	

Telangiectasia (lower eyelid)	A	2 (1, 2)	1 (1, 1)	1 (1, 1)	0.102
	B	1 (1, 2)	1 (1, 2)	1 (1, 2)	0.345
	*p* ^#^	0.281	0.544	0.166	

^∗^ *p* value of data for each group at different time points.

^#^ *p* value of Groups A and B at a specific time point.

^a^ *p* < 0.05 vs. baseline.

^b^ *p* < 0.05 vs. 2 weeks after treatment.

### 3.3. Secondary Efficacy Index

Statistically significant improvements were observed in both groups for meibomian gland expressibility, MQ score, FBUT, and CFS score. Specifically, Group A exhibited a significantly lower MQ score compared to Group B. However, no significant differences were observed between the two groups for the remaining parameters. Additionally, no significant differences were detected in TMH and LLT between the groups (Table 3 and Figure S2).

**TABLE 3:** The secondary efficacy index before and after treatment.

Index	Group	Baseline	Week 2	Week 4	*p* ^∗^
Meibomian gland expressibility (upper eyelid)	A	1 (1, 2)	1 (1, 1)^a^	1 (0, 1)^ab^	< 0.001
	B	1 (1, 2)	1 (1, 1)	1 (0, 1)^ab^	0.002
	*p* ^#^	0.493	0.445	0.609	

Meibomian gland expressibility (lower eyelid)	A	1 (1, 2)	1 (0, 1)^a^	0 (0, 1)^ab^	< 0.001
	B	1 (1, 2)	1 (0, 1)	1 (0, 1)^a^	0.004
	*p* ^#^	0.556	0.718	0.268	

MQ score (upper eyelid)	A	2 (1, 2)	1 (0, 2)^a^	1 (0, 1)^ab^	< 0.001
	B	2 (2, 2)	2 (1, 2)^a^	1 (1, 2)^a^	< 0.001
	*p* ^#^	0.080	0.020	0.001	

MQ score (lower eyelid)	A	1 (1, 1)	1 (1, 1)^a^	1 (0, 1)^a^	< 0.001
	B	2 (1, 2)	1 (1, 2)	1 (1, 1)^b^	0.001
	*p* ^#^	0.203	0.005	0.021	

FBUT (s)	A	3.53 ± 1.74	4.83 ± 2.39^a^	4.70 ± 1.83^a^	0.005
	B	3.70 ± 1.63	4.73 ± 1.38^a^	4.49 ± 1.82	0.011
	*p* ^#^	0.731	0.867	0.689	

CFS score	A	2.92 ± 1.26	1.81 ± 0.98^a^	0.85 ± 0.78^ab^	< 0.001
	B	2.80 ± 1.08	2.48 ± 1.23	1.00 ± 0.76^ab^	< 0.001
	*p* ^#^	0.710	0.035	0.482	

TMH (mm)	A	0.19 ± 0.06	0.19 ± 0.06	0.18 ± 0.04	0.436
	B	0.23 ± 0.12	0.22 ± 0.10	0.21 ± 0.04	0.372
	*p* ^#^	0.074	0.057	0.068	

LLT (nm)	A	75.15 ± 22.32	71.35 ± 21.98	75.46 ± 23.83	0.613
	B	80.20 ± 21.19	78.32 ± 26.61	81.44 ± 19.51	0.744
	*p* ^#^	0.412	0.312	0.333	

^∗^ *p* value of data for each group at different time points.

^#^ *p* value of Groups A and B at a specific time point.

^a^ *p* < 0.05 vs. baseline.

^b^ *p* < 0.05 vs. 2 weeks after treatment.

### 3.4. Safety Assessment

BCVA remained unchanged throughout the treatment period. IOP increased slightly at 2 weeks posttreatment but remained within the normal range (Table 4). At 4 weeks, IOP decreased and showed no significant difference compared to baseline. During the procedure, approximately 30% of patients reported itching of the eyelid margins, which resolved spontaneously shortly after treatment. No serious adverse events such as corneal or skin injury or vision loss occurred in anyone.

**TABLE 4:** The safety assessment before and after treatment.

Index	Group	Baseline	Week 2	Week 4	*p* ^∗^
BCVA	A	0.66 ± 0.36	0.68 ± 0.36	0.69 ± 0.36	0.107
	B	0.63 ± 0.33	0.60 ± 0.32	0.60 ± 0.32	0.067
	*p* ^#^	0.777	0.399	0.331	

IOP (mmHg)	A	12.62 ± 2.54	13.05 ± 2.43^a^	12.94 ± 2.21	0.035
	B	12.94 ± 2.27	13.43 ± 2.12^a^	13.27 ± 2.07	0.018
	*p* ^#^	0.633	0.557	0.589	

^∗^ *p* value of data for each group at different time points.

^#^ *p* value of Groups A and B at a specific time point.

^a^ *p* < 0.05 vs. baseline.

## 4. Discussion

Blepharitis is a chronic ocular inflammation mainly involving the eyelid margin and is a common cause of chronic ocular irritation. The pathogenesis of blepharitis is complex. Among them, an increase of local bacterial load and the change of microbiota composition have important influences on the recurrence of blepharitis and its long‐term impact on the life of patients [31]. For patients with blepharitis, reducing the bacterial load and cleaning the eyelid margin are crucial for the improvement of the disease. This prospective, randomized controlled study assessed the efficacy of the Deep Cleaning Device as a treatment for blepharitis by employing distinct treatment approaches, with a particular emphasis on their effects on bacterial load and clinical symptoms and signs.

This study demonstrated that the Deep Cleaning Device significantly reduces bacterial load and alleviates subjective symptoms in patients with blepharitis. This finding is largely consistent with previous research on eyelid margin cleaning [19, 25]. Specifically, Group A, which received treatment with eye drops combined with weekly eyelid margin deep cleaning, exhibited marked improvements in bacterial load and subjective symptoms. These improvements were more pronounced as the treatment duration increased, indicating the device’s effectiveness in managing blepharitis over time. Additionally, objective signs, including eyelid margin scale grading, showed significant improvements in Group A compared to Group B, which received eye drops alone. However, no significant differences were observed in telangiectasia between the two groups before and after treatment. This may be attributed to the relatively short treatment period, suggesting that a longer intervention might be necessary to achieve a more pronounced effect on telangiectasia. Additionally, this finding could also be related to the nature of blepharitis itself, which is primarily characterized by increased scales and microbiota dysbiosis, resulting in a relatively low telangiectasia score at baseline. The observed reductions in bacterial load and clinical symptoms in Group A suggest that the Deep Cleaning Device effectively removes harmful bacteria from the eyelid margins, thereby reducing inflammation and improving overall ocular health. This finding is consistent with previous studies highlighting the role of bacterial overgrowth in the pathogenesis of blepharitis [11, 31, 32]. By mechanically removing bacteria and scales, the Deep Cleaning Device addresses a key factor contributing to the condition, leading to symptom relief and improved clinical outcomes.

Beyond the primary efficacy indices, secondary efficacy indices such as meibomian gland expressibility, MQ, FBUT, and CFS score also showed improvements in both groups. Among them, there was a significant difference in MQ between the two groups, and Group A was superior to Group B. This indicates that deep cleaning is beneficial for improving MQ. This has not been reported in previous studies. Some studies, after conducting lipidomics analysis of MGD in patients with MGD by liquid chromatography‐mass spectrometry, found that the properties of meibomian esters are related to lipid composition [33, 34]. In patients with blepharitis, the abundance of *Staphylococcus* tends to rise, and the local bacterial metabolism can generate lipase, which in turn modifies the composition of meibum [32, 35]. The enhancement in MQ observed in this study may be linked to the effective suppression of bacterial proliferation at the eyelid margin achieved through deep cleaning. Although there was no significant difference in meibomian gland expressibility, FBUT, and CFS score between the two groups, Group A was generally better than Group B, and studies with larger sample sizes may be needed to further elucidate the impact of deep cleaning on these parameters. There were no significant changes in TMH and LLT before and after treatment. This might be due to the fact that the lacrimal gland secretion ability of patients with blepharitis was not severely impaired, or it might be because the observation time was relatively short.

Safety assessments revealed no significant changes in BCVA or IOP in either group, indicating that the Deep Cleaning Device is safe for use in patients with blepharitis. The slight increase in IOP observed at 2 weeks posttreatment in both groups remained within the normal range and was not clinically significant. By Week 4, IOP had returned to baseline levels, further supporting the safety profile of the device. Only 30% of the patients reported itching during the treatment, and the symptoms resolved after the treatment. This might be related to the mild friction of the skin caused by the high‐speed rotation of the brush head. No patients presented with serious adverse reactions such as decreased vision and corneal or skin abrasions.

This study has several limitations. First, due to the nature of the intervention, subjects in Group A were aware that they were receiving the additional deep cleaning treatment, which introduces a potential performance bias. Therefore, the potential placebo effect and participant bias cannot be ignored. Second, the clinical follow‐up duration may have been insufficient to evaluate long‐term therapeutic outcomes. Future investigations should incorporate (1) extended longitudinal observation periods, (2) larger multicenter cohorts to enhance statistical power, (3) sham‐controlled study arms to isolate treatment‐specific effects, and (4) metagenomic sequencing to characterize treatment‐associated microbiome alterations.

## 5. Conclusions

In conclusion, this study demonstrates that the Deep Cleaning Device is a safe and effective treatment for blepharitis, significantly reducing bacterial load and improving MQ. Its integration into treatment protocols could enhance patient outcomes and contribute to the management of this challenging condition.

## Author Contributions

Lei Tian, Ying Jie, and Ya Wen designed the experiments. Ya Wen, Jiayu Bao, and Ao Li conducted the experiments and wrote the paper. Yubo Wu and Yang Zhang helped conduct the experiments and data analysis. Lei Tian and Ying Jie supervised the work and provided financial support.

## Funding

This research was supported by the Talent Development Plan for High‐Level Public Health Technical Personnel Project, Academic Leaders, 02‐22; Beijing Municipal Public Welfare Development and Reform Pilot Project for Medical Research Institutes (PWD&RPP‐MRI, JYY2023‐6); Capital Health Research and Development of Special (Grant No. 2022‐1‐1081 to Dr. Ying Jie); Beijing Hospitals Authority’s Ascent Plan (DFL20240202); Youth Beijing Scholars Program (No. 022); National Natural Science Foundation of China (81970764); and Yicheng Outstanding Talents program from the Beijing Economic–Technological Development Area.

## Ethics Statement

This study was approved by the Ethics Review Committee of Beijing Tongren Hospital (Ethics Approval Number: TRECKY2021‐065). This study was enlisted in the Chinese Clinical Trial Registry.

## Conflicts of Interest

The authors declare no conflicts of interest.

## Supporting Information

Additional supporting information can be found online in the Supporting Information section.

## Supporting information


**Supporting Information** This document contains the following supporting figures. Figure S1: The primary efficacy index before and after treatment. Figure S2: The secondary efficacy index before and after treatment.

## Data Availability

All data generated or analyzed during this study are included in this published article/as supporting information files.
