# Supplementary material for: Deep Cleaning Device as a Treatment Is Effective for Blepharitis and Significantly Reduces Bacterial Load
Source: J Ophthalmol. 2026 Jun 2;2026:1518139. doi: 10.1155/joph/1518139 (PMC13239489; doi:10.1155/joph/1518139)
Supplement: Supplementary file 1 — Supporting Information This document contains the following supporting figures. Figure S1: The primary efficacy index before and after treatment. Figure S2: The secondary efficacy index before and after treatment. [file JOPH-2026-1518139-s001.docx]

**Supplementary figures:**
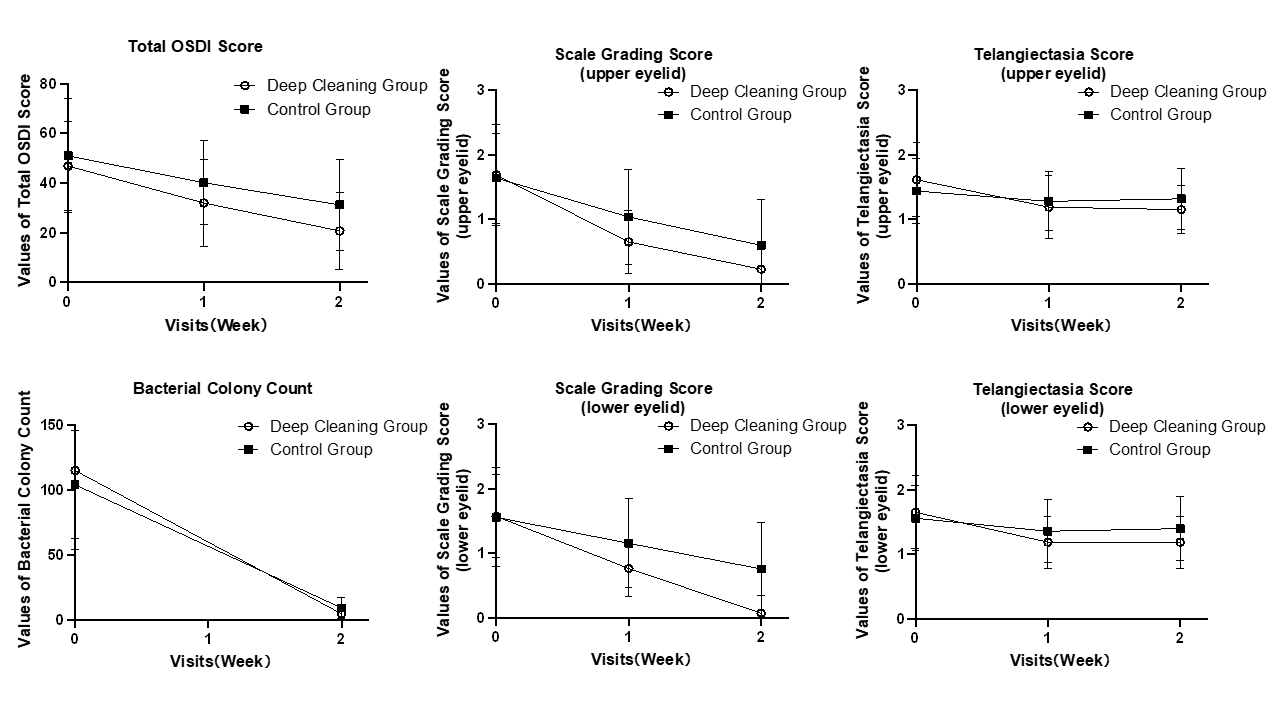


**Figure S1**. The primary efficacy index before and after treatment. Line graphs show the changes in the primary efficacy index between baseline and post-treatment time points. Data are presented as mean ± SD.


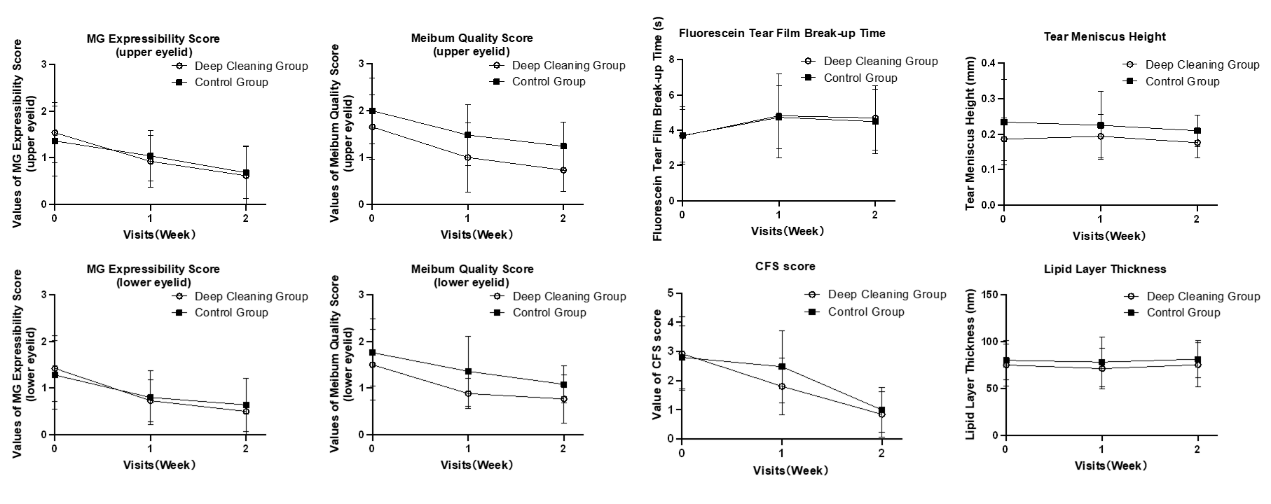


**Figure S2**. The secondary efficacy index before and after treatment. The comparison of the secondary efficacy index at baseline and after treatment is shown. Data are presented as mean ± SD.
